# Resection of Superior-Third of Humerus

**Published:** 1864-08

**Authors:** F. Marion Letcher

**Affiliations:** Assistant Surgeon P. A. C. S.


ART. V.—*Resection of Superior Third of Humerus.* By F. MARION LETCHER, Assistant Surgeon P. A. C. S.

The following case, which came under my care while connected with the General Hospital, Liberty, Virginia, is supposed to be of sufficient interest to report:

Private Harvey Barton, company "D," 28th Virginia regiment of infantry, age 27, stout, healthy, a farmer, was accidentally wounded in left shoulder, November 11th, 1863, by the discharge of a musket in his own hands.

Wound inflicted by minie balll passing through tendon of pectoralis-major muscle beneath anterior border of deltoid muscle, and escaping through posterior portion of this muscle just below posterior margin of the acromion process. The muzzle of the gun being but a few inches from the point struck, the ball made an immense track, greatly lacerating the soft parts, and leaving a hiatus in the bone at least two inches long.

There was considerable hemorrhage, which proceeded from some of the smaller vessels about the joint—probably the anterior or posterior circumflex artery. This was soon arrested by position and the application of cold water. The shock was very great.

Re-action having taken place, complete anæsthesia was produced by the inhalation of chloroform, twenty-six hours after injury.

The wound was now carefully examined and a consultation held, the result of which was, that resection of the humerus should be performed, rather than amputation at the shoulder-joint.

*Operation.*—A semi-lunar incision was made through the deltoid muscle, connecting wounds of entrance and exit. This done and the deltoid raised, the whole extent of the wound was exposed, the limb dangling and being supported only by the triceps lacerated, the coraco-brachialis and the short head of the biceps. Between these muscles, were the larger vessels and nerves of the part, uninjured.

A longitudinal fracture was found, passing through the head of the humerus; this was now carefully dissected from its glenoid cavity.

The arm was now carried transversely across the thorax, all the detached fragments of bone removed, the dissection made as far downwards as the communication extended, and the bone sawn across four inches from the anatomical neck. Fifteen fragments of bone were taken away. The wound, cleansed of partially detracted portions of lacerated tissues, was closed and supported by sutures. The limb, gently pressed upwards by bandage at the elbow, was placed in a comfortable position, and cold-water dressings constantly applied to the wound. Suppuration was almost entirely absent. The deltoid united by first intention. The patient had a rapid recovery; there was not an untoward symptom.

*Present Condition.*—The limb is shortened two inches—movements of forearm unimpaired; those of arm, of course, not perfect, but all partially made, and even with considerable facility when patient makes a great effort. Within the last two months, there has been very marked improvement in this respect, and it is believed that by constant exertion, the limb will be restored to almost its former mobility.

*Remarks.*—The operation in which the deltoid muscle is divided, has been objected to, *because its functions are permanently destroyed.* Probably it is Mr. Guthrie who was first to refute this objection, by showing that division of this muscle does not necessarily lead to permanent disuse, but that under favorable circumstances perfect usefulness may be regained. Certainly, but few cases, demanding this operation, will ever be presented to the surgeon more unpromising than was the one which is the subject of this report. And, yet, a limb is preserved whose functions are already partially performed, with flattering indications of speedy and permanent usefulness.

